# Total Lung and Lobar Quantitative Assessment Based on Paired Inspiratory–Expiratory Chest CT in Healthy Adults: Correlation with Pulmonary Ventilatory Function

**DOI:** 10.3390/diagnostics11101791

**Published:** 2021-09-28

**Authors:** Feihong Wu, Leqing Chen, Jia Huang, Wenliang Fan, Jinrong Yang, Xiaohui Zhang, Yang Jin, Fan Yang, Chuansheng Zheng

**Affiliations:** 1 Department of Radiology, Union Hospital, Tongji Medical College, Huazhong University of Science and Technology, 1277 Jiefang Rd., Wuhan 430022, China; wfh_wuhan@hust.edu.cn (F.W.); leqingchen@hust.edu.cn (L.C.); huangjiapal@hust.edu.cn (J.H.); fwl@hust.edu.cn (W.F.); yangjr@hust.edu.cn (J.Y.); 2 Hubei Province Key Laboratory of Molecular Imaging, Wuhan 430022, China; 3 Clinical Science, Philips Healthcare, No. 718 Daning Rd., Jingan District, Shanghai 200233, China; xiaohui.zhang_1@philips.com; 4 Department of Respiratory and Critical Care Medicine, Union Hospital, Tongji Medical College, Huazhong University of Science and Technology, 1277 Jiefang Rd., Wuhan 430022, China; whuhjy@126.com

**Keywords:** tomography, X-ray computed, quantitative analysis, pulmonary function test, ventilation

## Abstract

Objective: To provide the quantitative volumetric data of the total lung and lobes in inspiration and expiration from healthy adults, and to explore the value of paired inspiratory–expiratory chest CT scan in pulmonary ventilatory function and further explore the influence of each lobe on ventilation. Methods: A total of 65 adults (29 males and 36 females) with normal clinical pulmonary function test (PFT) and paired inspiratory–expiratory chest CT scan were retrospectively enrolled. The inspiratory and expiratory volumetric indexes of the total lung (TL) and 5 lobes (left upper lobe [LUL], left lower lobe [LLL], right upper lobe [RUL], right middle lobe [RML], and right lower lobe [RLL]) were obtained by Philips IntelliSpace Portal image postprocessing workstation, including inspiratory lung volume (LV_in_), expiratory lung volume (LV_ex_), volume change (∆LV), and well-aerated lung volume (WAL, lung tissue with CT threshold between −950 and −750 HU in inspiratory scan). Spearman correlation analysis was used to explore the correlation between CT quantitative indexes of the total lung and ventilatory function indexes (including total lung capacity [TLC], residual volume [RV], and force vital capacity [FVC]). Multiple stepwise regression analysis was used to explore the influence of each lobe on ventilation. Results: At end-inspiratory phase, the LV_in-TL_ was 4664.6 (4282.7, 5916.2) mL, the WAL_TL_ was 4173 (3639.6, 5250.9) mL; both showed excellent correlation with TLC (LV_in-TL_: *r* = 0.890, *p* < 0.001; WAL_TL_: *r* = 0.879, *p* < 0.001). From multiple linear regression analysis with lobar CT indexes as variables, the LV_in_ and WAL of these two lobes, LLL and RUL, showed a significant relationship with TLC. At end-expiratory phase, the LV_ex-TL_ was 2325.2 (1969.7, 2722.5) mL with good correlation with RV (*r* = 0.811, *p* < 0.001), of which the LV_ex_ of RUL and RML had a significant relationship with RV. For the volumetric change within breathing, the ∆LV_TL_ was 2485.6 (2169.8, 3078.1) mL with good correlation with FVC (*r* = 0.719, *p* < 0.001), moreover, WAL_TL_ showed a better correlation with FVC (*r* = 0.817, *p* < 0.001) than that of ∆LV_TL_. Likewise, there was also a strong association between ∆LV, WAL of these two lobes (LLL and RUL), and FVC. Conclusions: The quantitative indexes derived from paired inspiratory–expiratory chest CT could reflect the clinical pulmonary ventilatory function, LLL, and RUL give greater impact on ventilation. Thus, the pulmonary functional evaluation needs to be more precise and not limited to the total lung level.

## 1. Introduction

Chest computed tomography (CT) is a well-known imaging method for displaying the pulmonary morphological state for qualitative evaluation; it also has the potential to reflect the histopathological or functional status combined with quantitative analysis. For example, the area where the CT value lower than −950 hounsfield units (HU) at end-inspiratory scanning is considered as emphysema tissue, which has been confirmed by histopathology [1]; the area below −856HU at end-expiratory scanning is considered as air trapping [2,3]; also, a quantitative CT can be used to evaluate small airway diseases when combined with airway-wall measurements so as to explain the presence of respiratory symptoms beyond the information offered by the clinical pulmonary function test (PFT) [4,5]. In addition, the paired inspiratory–expiratory chest CT has been studied in chronic obstructive pulmonary disease (COPD), which is characterized by incompletely reversible airflow limitation for further exploration [6,7]. However, the previous quantitative CT studies were consistent with PFT that mostly remained at the total-lung level. Zach et al. [8] reported that the volume change and mean lung density of upper lobes and lower lobes within a respiratory cycle were different, which suggests that the pulmonary functional imaging is far from enough at the total-lung level. It is feasible to segment and analyze the pulmonary lobe independently with the fast development of high-resolution CT and image postprocessing technology.

At present, there are few baseline studies of paired inspiratory–expiratory chest CTs in healthy subjects, mainly limited by the additional radiation this technique brought. Recent advances in CT equipment and the promotion of low-dose CT, which has controlled CT acquisition at significantly lower radiation doses within a safe range [9,10]. For the target population, during the post-COVID-19 (coronavirus disease 2019) stage, the public is under varying degrees of psychological stress, especially in severely infected areas, regardless of whether they were infected with SARS-CoV-2 [11,12]. In this environment, people who are very concerned about their pulmonary function will seek medical help, and doctors may recommend PFT and/or paired inspiratory–expiratory chest CT scan to evaluate the lung status after comprehensive consideration. This gives us the opportunity to obtain both types of examination from healthy people.

The purpose of this study is to provide quantitative paired inspiratory–expiratory chest CT data of the total lung and each lobe of healthy people and to demonstrate the relationship of indexes between CT and clinical PFT, then further explore the effect of lobes on ventilatory function, thus providing baseline data and basic information for quantitative CTs on pulmonary disease research in the future.

## 2. Materials and Methods

### 2.1. Study Subjects

Subjects with PFT and paired inspiratory–expiratory chest CT in our hospital from September 2020 to April 2021 were retrospectively collected. Inclusion criteria: (1) age older than 18 years; (2) all indexes of PFT were normal; (3) the PFT and CT scan were completed within 3 days. Exclusion criteria: (1) the CT images cannot be segmented by postprocessing because of motion artifacts; (2) pulmonary lesions were presented in CT images; (3) pulmonary lobe variants, thoracic deformities, or chest surgery history. The retrospective study was approved by the ethics committee of our hospital (No. 0271-01), and the informed consents were waived. A total of 65 adults with normal clinical pulmonary function who met the above-mentioned criteria were enrolled (Table 1), including 29 males and 36 females, median age, 56 (43, 63) years old.

### 2.2. Pulmonary Function Tests

Ventilatory function (including spirometry and lung volume) was measured in all subjects using a flow spirometer (MasterScreen; CareFusion, Hoechberg, Germany) by trained technicians according to the guidelines [13,14]. The following indexes were recorded: (1) total lung capacity (TLC), which refers to the volume of gas within the lungs after maximal inspiration; (2) residual volume (RV), which refers to the volume of gas remaining in the lung after maximal expiration; and (3) forced vital capacity (FVC), which refers to the volume of gas that is exhaled during a forced expiration that is starting from full inspiration and ending at complete expiration.

### 2.3. Chest CT Scan

All CT scans were performed with a 64-detector scanner (IQon Spectral CT, Philips Healthcare, Best, The Netherlands), the scanning range was from the inlet of thorax to the level of the adrenal glands at full inspiration and repeated at full expiration with the subjects in the supine position. Before scanning, each subject was carefully trained on how to breathe during scanning by an experienced technician (W.L.F. with 11 years of experience). Scanning voltage was 120 kV, and 3D tube current automatic modulation technology was used, the pitch was 0.984, and the detector was 64 × 0.625 mm. The volume computed tomography dose index (CTDI_vol_) was 5.9 ± 1.3 mGy (range: 3.4–7.5 mGy) per scan. All imaging was performed with a standard reconstruction algorithm with a slice thickness of 1.0 mm and a reconstruction interval of 0.5 mm.

### 2.4. Imaging Segmentation and Quantitative Measurements

**Figure 1:**
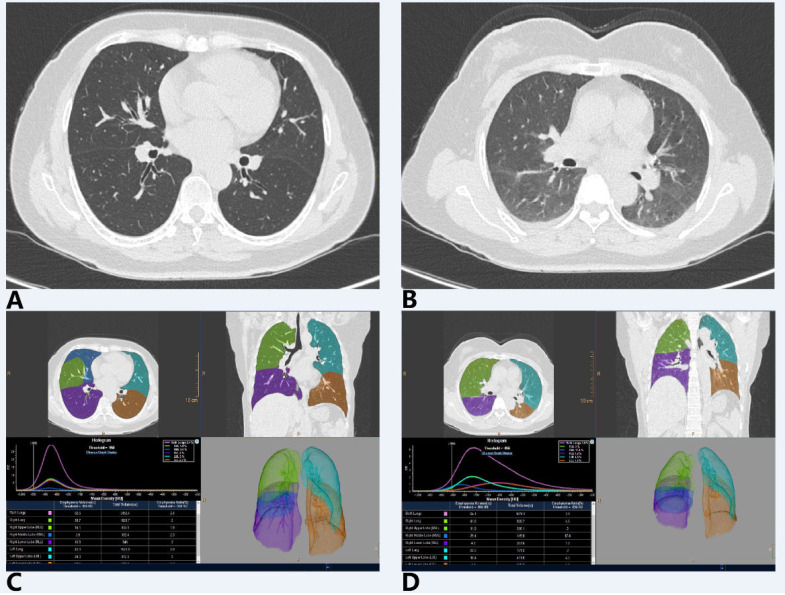
Paired inspiratory–expiratory chest CT image and postprocessing interface. (**A**) Chest CT image at deep-inspiratory phase; (**B**) Chest CT image at deep-expiratory phase; (**C**, **D**) Postprocessing and quantitative analysis on inspiratory and expiratory chest CT images.

The CT images in DICOM format were sent to the Philips IntelliSpace Portal postprocessing workstation (version 12.0) and were performed with Chronic Obstructive Pulmonary Disease (COPD) analysis software (Figure 1). Each postprocessing step was as follows: firstly, define the trachea–bronchial tree and automatically extract it out; secondly, identify the left and right lung contours, and then identify the interlobular fissures to divide the five lobes (left upper lobe [LUL], left lower lobe [LLL], right upper lobe [RUL], right middle lobe [RML], and right lower lobe [RLL]) by automatic segmentation, manual corrections were performed when segmentation was inaccurate. The CT value threshold was set as −950 HU to −750 HU in end-inspiratory phase to obtain the well-aerated lung. The following quantitative CT indexes of the total lung (TL) and 5 lobes were recorded: In the inspiratory phase, the inspiratory lung volume (LV_in_), the well-aerated lung volume (WAL), and the inspiratory mean lung density (MLD_in_) were obtained; in the expiratory phase, the expiratory lung volume (LV_ex_) and expiratory mean lung density (MLD_ex_) were obtained. The difference between the LV_in_ and LV_ex_ was defined as lung volume change (∆LV).

### 2.5. Statistical Analysis

**Figure 2:**
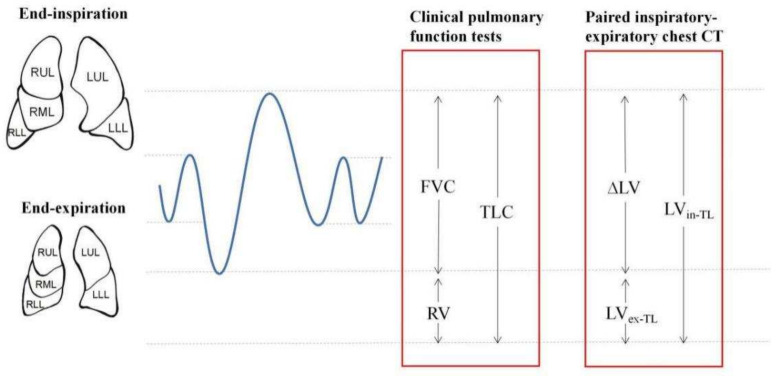
Schematic diagram of ventilatory function and paired inspiratory–expiratory chest CT indexes during breathing. The blue line represents the lung volumes and capacities on static breathing and full inspiration-expiration.

SPSS 26.0 software (IBM, New York, NY, USA) was used for statistical analysis. All data were represented by the median (Q1, Q3) or *n/N* (%). Considering the examination processing similarity between paired inspiratory–expiratory chest CT and PFT, the relationship between the ventilation indexes and the CT quantitative indexes in corresponding phase (inspiration, expiration, and the volume change by breathing, respectively, as shown in Figure 2) were analyzed, and the analysis on WAL was also included: LV_in_ & TLC, WAL & TLC, LV_ex_ & RV, ∆LV & FVC, and WAL & FVC. Spearman correlation analysis was used to explore the correlation between CT quantitative indexes of the total lung and ventilatory function indexes. To simplify analysis of lobar differences, we divided lobar indexes into upper lobes (including the bilateral upper lobe and RML for its homologousness with the lingual lobe of left upper lobe: LUL + RUL + RML) and lower lobes (including the bilateral lower lobe: LLL + RLL). The Mann-Whitney *U* test was used to compare the difference of the volumetric CT indexes of upper lobes and lower lobes. Furthermore, multiple linear regression analysis was used to explore the correlation of each lobe on ventilation indexes (for TLC, RV, and FVC, respectively): lobar CT indexes were confirmed to be significant by performing stepwise methods, variables remained with an entry criterion of *p* < 0.05 and a removal criterion of *p* > 0.10. *p* values < 0.05 were considered statistically significant.

## 3. Results

### 3.1. Correlation Analysis between Quantitative CT and Ventilatory Function Indexes

**Figure 3:**
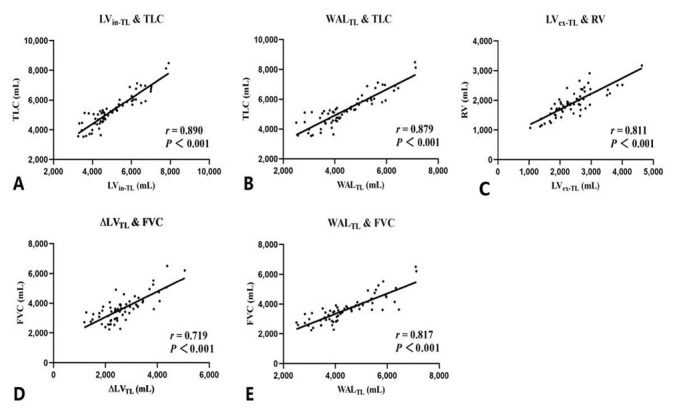
(**A**) the correlation between the inspiratory total lung volume (LVin-TL) and total lung capacity (TLC), *r* = 0.890, *p* < 0.001; (**B**) the correlation between the total well-aerated lung volume (WALTL) and TLC, *r* = 0.879, *p* < 0.001; (**C**) the correlation between the expiratory total lung volume (LVex-TL) and residual volume (RV), *r* = 0.811, *p* < 0.001; (**D**) the correlation between the total lung volume change (ΔLVTL) and forced vital capacity (FVC), *r* = 0.719, *p* < 0.001; (**E**) the correlation between WALTL and FVC, *r* = 0.817, *p* < 0.001.

The paired inspiratory–expiratory chest CT quantitative results of the total lung are shown in Table 2, the correlation analysis between quantitative CT indexes of total lung and ventilatory function are shown in Figure 3. In the inspiratory phase, the total lung density decreased due to the expansion of lung tissue and the inhalation of gas (gas presented as very low attenuation on CT), the MLD_in-TL_ (−843.8 [−853, −830.8] HU) was lower than MLD_ex-TL_ (−689.9 [−732.5, −663.1] HU).

At the end-inspiratory phase, LV_in-TL_ (4664.6 [4282.7, 5916.2] mL) showed an excellent correlation with TLC (5170.0 [4570.0, 5890.0] mL), *r* = 0.890, *p* < 0.001. Similarly, WAL_TL_ (4173.0 [3639.6, 5250.9] mL) represented part of the lung tissue measured in inspiration, it also presented a good correlation with TLC *(r* = 0.879, *p* < 0.001), but it was slightly lower than that of LV_in-TL_. At the end-inspiratory phase, LV_ex-TL_ (2325.2 [1969.7, 2722.5] mL) showed an excellent correlation with RV (1870.0 [1680.0, 2170.0] mL) with *r* = 0.811, *p* < 0.001. For the volume change between inspiration and expiration, ∆LV_TL_ (2485.6 [2169.8, 3078.1] mL) had good correlation with FVC (3580.0 [2990.0, 3930.0] mL) with *r* = 0.719, *p* < 0.001. Moreover, WAL_TL_ represented well-aerated lung tissue and showed a better correlation than ∆LV_TL_ and FVC (*r* for WAL_TL_ and FVC = 0.817, *p* < 0.001).

### 3.2. Multiple Linear Regression Analysis

**Figure 4:**
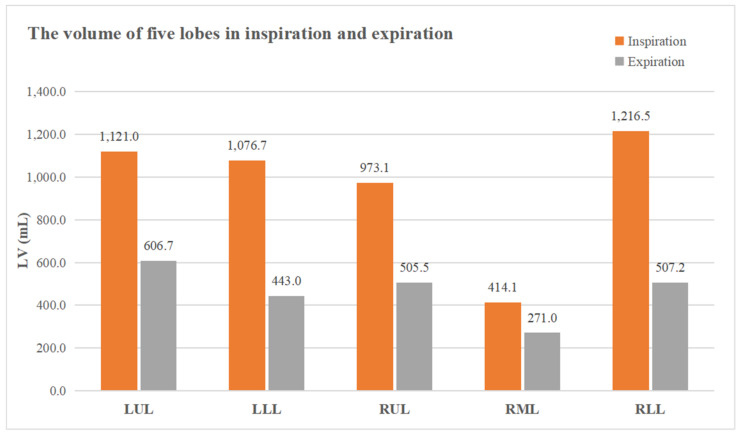
The lobar volume measured by paired inspiratory–expiratory chest CT.

The paired inspiratory–expiratory chest CT quantitative results of each lobe are shown in Table 2 and Figure 4, multiple linear regression analysis for lobar quantitative CT indexes on ventilatory function are shown in Table 3. All the quantitative volumetric CT indexes between upper lobes (LUL + RUL + RML) and lower lobes (LLL + RLL) were different (all *p* < 0.001): except for ∆LV, the other three indexes (LV_in_, LV_ex_ and WAL) were all larger in the upper lobes than in the lower lobes. It indicated that, although the lower lobes occupy a smaller proportion of the total lung within the whole breathing circle, the volumetric change degree was larger.

The contribution of each lobe to ventilatory function was analyzed by multiple linear regression. For TLC, from the LV_in_ and WAL of the five lobes, the CT quantitative indexes derived from LLL and RUL gave strong correlation to TLC (all *p* < 0.05). Similarly, for FVC, there was a strong association between ∆LV, WAL of these two lobes (LLL and RUL), and FVC (all *p* < 0.05). However, the difference was that through the analysis of LVex and RV, only the upper lobes of the right lung (RUL and RML) were associated with RV.

## 4. Discussion

In the past few decades, the most clinically used methods for pulmonary functional imaging have been nuclear medicine methods with gaseous radionuclides [15]. With the advancement of CT equipment and supporting software, CT images can not only provide qualitative information for routine subjective diagnosis, but also provide rich quantitative information from axial two-dimensional to three-dimensional structure, which can broaden the view for pulmonary function evaluation [16,17]. There have been studies of quantitative CT on assessment of air trapping in small airway diseases, as well as for assessing COPD phenotype [6,18,19]. However, there is a lack of baseline quantitative chest CT studies in healthy people. Our study focused on adults with normal clinical pulmonary function, who revealed good correlation on indexes between paired inspiratory–expiratory chest CT and clinical pulmonary ventilatory function. Furthermore, we show the importance of evaluation on the lobar level for ventilatory function, which offers novel insights on pulmonary functional imaging.

This baseline study demonstrates the feasibility of paired inspiratory–expiratory chest CT in the evaluation of pulmonary ventilatory function as multiple pairs of correlation coefficient indexes were >0.8 (Figure 2). In terms of the examination process, compared with PFT, the CT scan is more tolerant to the subject’s cooperation, and the operation by medical technicians is relatively simple and quick. Subjects only need to undergo simple pre-examination training and follow relevant instructions. However, the radiation exposure caused by paired inspiratory and expiratory CT cannot be ignored. Reduced radiation doses have been successfully used in various pulmonary disorders, and low-dose CT can eliminate this concern and control the radiation dose within a safe range while the image quality can meet the needs of diagnosis and postprocessing [7,9]. In terms of the organ (lung) being evaluated, PFT is currently considered as the gold standard for the pulmonary functional evaluation, however, it is insensitive to regional alternations and weak for morphological evaluation. It may indicate lesions indirectly with abnormal function caused by pulmonary structural changes. In contrast, the chest CT can visualize lesions noninvasively, and even demonstrate the functional unit of lung, the secondary pulmonary lobules in millimeter-scale images, and it has the potential to further present micro-scale anatomy in the future [20].

Our study showed that although there were good correlations between indexes of these two tests at corresponding breathing phase, the actual values were different. For PFT, the residual volume that cannot be directly detected by the spirometer needs to be converted indirectly by nitrogen washout or plethysmography [13,14], including RV, FRC, and TLC. These generated results include the volume of air within the trachea–bronchial tree, which may overestimate the actual ventilatory function of the lung. In the paired inspiratory–expiratory chest CT, the postprocessing software could automatically extract and remove the air within trachea–bronchial tree, thus a more accurate quantitative value of total lung volume can be obtained than TLC measured by PFTs. However, current quantitative indexes derived by noncontrast enhanced CT could only interpret ventilation and air trapping, which may not reflect the perfusion function as alveolar-capillary gas exchange that is defined as the process of molecular oxygen diffused into the plasma and red blood cell and carbon dioxide left from the plasma to the alveolar space. Nonetheless, the four-dimensional computed tomography (4DCT) with density-change-based methods reported by Castillo et al. [21] may have the ability to reflect true perfusion. Thus, the application of quantitative CT in pulmonary function deserves further studies.

Gattinoni et al. [22] divided lung tissues into different ventilatory functions as nonaerated, poorly aerated, normally aerated, and hyperinflated according to different CT threshold values. We selected the CT threshold value between −950 and −750 HU at end-inspiratory phase as well-aerated lung based on previous pulmonary quantitative studies on smokers and COVID-19 patients [23,24]. Our results showed a strong correlation between WAL_TL_ and TLC (*r* = 0.879), possibly because the WAL_TL_ also represents a large proportion of lung tissue in healthy people. Moreover, it is worth noting that WAL_TL_ showed a better correlation than ∆LV_TL_ and FVC when referring to the volume change assessment (*r* for WAL_TL_ and FVC = 0.817, *r* for ∆LV and FVC = 0.719). It demonstrates that, on the one hand, WAL may be a well indicator to reflect both total lung volume and vital capacity, and on the other hand, it also indicates the feasibility of quantitative indexes derived from a single-phase CT scan for the dynamic vital capacity evaluation. When the lung is under a pathological state, part of the lung tissue may occupied by lesions, such as mucus or inflammation. It will inevitably lead to a decrease or loss of local ventilation [25]. It is possible to distinguish the proportion of lesion areas by setting different CT threshold values under these conditions, such as ground-glass opacity (−750~−300 HU) and consolidation (>−300HU) [26,27], rather than being limited to subjective qualitative assessment. Therefore, it is necessary to broaden the exploration and understanding of CT quantitative indexes derived from different threshold values on pulmonary functional imaging.

The pulmonary ventilation and perfusion are gravitational dependent, the upper lobes are more well ventilated and less perfused, whereas the lower lobes are better perfused and less ventilated in the upright position [28,29]. The CT scans in our study were performed in the supine position, which can balance the influence of gravity on upper and lower lung field. We further analyzed the effect of each lobe on pulmonary ventilatory function, and it revealed that there was a strong correlation from CT quantitative indexes of LLL and RUL through multiple linear regression analysis on pulmonary ventilatory function. Qi et al. [30] simulated the pulmonary airflow using full-inhaled CT image based models with computational fluid dynamics method, they demonstrated that the central flow enters the bilateral lower lobes with larger wall shear stress in healthy subjects, and lobar volumetric distribution of LLL was the most among five lobes (22.7–30.2% of the total lung volume). Our study on quantitative paired inspiratory–expiratory chest CT shows, although the volume of the upper lobes (LUL + RUL + RML) were larger than that of the lower lobes (LLL + RLL) both at the end of inspiration and expiration (Table 2), the effect of LLL on ventilatory function cannot be ignored since the indexes of LLL were repeatedly selected out by multiple linear regression analysis. In addition, the airflow and gas distribution in RUL are not dominant by fluid dynamics analysis, our results presented that, unexpectedly, the CT indexes from RUL screened out in each multiple linear regression analysis, which illustrates the extremely important role of RUL among the five lobes on pulmonary ventilatory function. Therefore, this indicates that the pulmonary function evaluation should not be limited to the total lung level due to the effect if each lobe on ventilation is not balanced. The clinical guiding significance points out that when lobectomy or lung volume reduction surgery involving RUL and LLL, ventilated dysfunction caused by lung tissue decreasing in these two regions should be considered carefully, rather than only focusing on the lesion clearance.

There were several limitations to this study. The quantitative CT results provided by this study were from Chinese people, and there may be differences in lung volume among different ethnic groups [31]. Due to the relatively small number of samples, the normal reference range of each CT indexes for healthy people and reference equations on predicting normal ventilatory function could not be established. In addition, CT image postprocessing relies on a good image quality, otherwise the postprocessing steps may not be be completed successfully or the quantitative CT results obtained are inaccurate due to motion artifacts. Besides, the quantitative parameters of airway wall thickness were not measured, mainly because the airway of healthy people in this study were regarded as without pathological changes, thus the relationship between the airway measurement and PFT indexes were not analyzed. Furthermore, the relationship between CT quantitative indexes and diffusion function needs to be studied in the future.

## 5. Conclusions

In conclusion, the paired inspiratory–expiratory chest CT has the potential to reflect the clinical pulmonary ventilatory function, and it could provide quantitative data on lobar volume with different threshold ranges. The evaluation of pulmonary function should not be limited to the total lung level, the two lobes, LLL and RUL, are essential for ventilatory function.

## Figures and Tables

**Table 1:** Demographic characteristics and pulmonary function results.

Characteristics	All Subjects (*n* = 65)
Demographic characteristics	
Age, years	56 (43, 63)
Sex	
Male, *n*/*N* (%)	29/65 (44.6%)
Female, *n*/*N* (%)	36/65 (55.4%)
Height, cm	163.0 (158.0, 169.0)
Weight, kg	64.0 (57.0, 72.0)
BMI, kg/m^2^	24.0 (22.7, 25.8)
PFT results	
Lung volume	
TLC, mL	5170.0 (4570.0, 5890.0)
TLC % predicted	99.1 (89.0, 105.4)
RV, mL	1870.0 (1680.0, 2170.0)
RV % predicted	99.7 (87.9, 108.3)
FVC, mL	3580.0 (2990.0, 3930.0)
FVC % predicted	109.6 (102.1, 122.6)
Spirometry	
FEV1 % predicted	100.9 (93.5, 115.0)
FEV1/FVC, %	78.1 (74.6, 81.6)
MEF_75%_ % predicted	107.5 (95.7, 121.7)
MEF_50%_ % predicted	80.5 (69.8, 100.1)
MMEF % predicted	78.0 (70.0, 90.8)

Note: Data are presented as median (Q1, Q3) or n/N (%). Abbreviations: PFT: pulmonary function test; BMI: body mass index; TLC: total lung capacity; RV: residual volume; FVC: forced vital capacity; FEV1: forced expiratory volume in one second; MEF: maximal expiratory flow; MMEF: maximal mid-expiratory flow.

**Table 2:** The paired inspiratory–expiratory chest CT quantitative results of the total lung and each lobe.

Indexes	TL	LUL	LLL	RUL	RML	RLL	Upper Lobes (LUL + RUL + RML)	Lower Lobes (LLL + RLL)	*p* Value
MLD_in_, HU	−843.8 (−853.0, −830.8)	−853.0 (−862.6, −842.1)	−825.8 (−840.5, −812.6)	−853.0 (−863.5, −844.5)	−854.4 (−868.1, −840.2)	−832.7 (−844.9, −820.1)	/	/	/
MLD_ex_, HU	−689.9 (−732.5, −663.1)	−716.9 (−759.8, −687.4)	−614.8 (−663.4, −570.5)	−742.8 (−765.8, −700.8)	−772.0 (−796.5, −750.5)	−638.9 (−677.8, −601.6)	/	/	/
LV_in_, mL	4664.6 (4282.7, 5916.2)	1121.0 (986.0, 1379.0)	1076.7 (915.4, 1327.4)	973.1 (809.2, 1143.1)	414.1 (386.9, 564.2)	1216.5 (1042.6, 1446.9)	2506.0 (2224.6, 3055.9)	2284.5 (1947.0, 2704.8)	<0.001
LV_ex_, mL	2325.2 (1969.7, 2722.5)	606.7 (474.8, 690.7)	443.0 (349.4, 521.0)	505.5 (419.4, 601.5)	271.0 (234.7, 332.6)	507.2 (430.9, 593.4)	1400.1 (1132.4, 1588.0)	942.1 (782.0, 1101.9)	<0.001
∆LV, mL	2485.6 (2169.8, 3078.1)	544.2 (457.9, 683.7)	674.8 (551.0, 790.7)	433.4 (333.7, 524.2)	170.1 (125.6, 219.2)	723.2 (604.3, 829.2)	1140.7 (972.0, 1397.8)	1381.5 (1118.7, 1602.6)	<0.001
WAL, mL	4173.0 (3639.6, 5250.9)	1007.0 (875.9, 1240.0)	939.7 (762.4, 1106.0)	864.7 (735.7, 1020.3)	370.7 (342.5, 501.6)	1050.7 (905.7, 1282.6)	2237.4 (1970.2, 2733.9)	2012.4 (1704.2, 2414.6)	<0.001

Note: Data are presented as the median (Q1, Q3). Abbreviations: MLD_in_: mean lung density in the inspiratory phase; MLD_ex_: mean lung density in the expiratory phase; LV_in_: lung volume in the inspiratory phase; LV_ex_: lung volume in the expiratory phase; ∆LV: lung (or lobar) volume change during respiration; WAL: well-aerated lung tissue volume; TL: total lung; LUL: left upper lobe; LLL: left lower lobe; RUL: right upper lobe; RML: right middle lobe; RLL: right lower lobe.

**Table 3:** Multiple linear regression analysis for ventilatory function.

	Estimate	Error	*t* Value	*p* Value
TLC				
LV_in_: (R^2^ = 0.809)				
LV_in-LLL_	1.401	0.263	5.331	<0.001
LV_in-RUL_	1.344	0.596	2.256	0.028
WAL: (R_2_ = 0.781)				
WAL_LLL_	1.613	0.257	6.265	0.001
WAL_RUL_	2.499	0.394	6.342	<0.001
RV				
LV_ex_: (R^2^ = 0.689)				
LV_ex-RUL_	1.377	0.250	5.502	0.001
LV_ex-RML_	1.083	0.508	2.133	0.037
FVC				
∆LV: (R^2^ = 0.576)				
∆LV_LLL_	2.024	0.364	5.568	0.001
∆LV_RUL_	1.724	0.72	2.394	0.020
WAL: (R^2^ = 0.677)				
WAL_LLL_	1.649	0.258	6.381	0.001
WAL_RUL_	1.257	0.396	3.176	0.002

Abbreviations: LV_in_: lung volume in the inspiratory phase; LV_ex_: lung volume in the expiratory phase; ∆LV: lung (or lobe) volume change during respiration; WAL: well-aerated lung tissue volume; TLC: total lung capacity; RV: residual volume; FVC: forced vital capacity; LLL: left lower lobe; RUL: right upper lobe; RML: right middle lobe.

## Data Availability

The data that support the findings of this study are available from the corresponding author upon reasonable request.
